# Beyond the biomedical: an evaluation of the introduction of social gerontology into a postgraduate geriatric medicine education program

**DOI:** 10.1007/s41999-023-00864-0

**Published:** 2023-10-05

**Authors:** Louise Tomkow, Alexander Thomson

**Affiliations:** 1 Humanitarian and Conflict Response Institute, University of Manchester, Ellen Wilkinson Building, Oxford Road, Manchester, M15 6JA UK; 2 Salford Care Organisation, Northern Care Alliance and University of Salford, Salford, UK

**Keywords:** Social Gerontology, Evaluation, Postgraduate education, Inequality

## Abstract

Geriatric Medicine education tends to adopt a biomedical lens, despite the practice of Geriatric Medicine involving the comprehensive assessment of the functional, psychological, and social aspects of older people’s lives. In this commentary, we describe the delivery of a Social Gerontology education program for Geriatric Medicine trainees in the Northwest of England. Education in Social Gerontology—a field that focuses on how social, cultural, economic, and environmental factors shape the lives of older adults—is thought to mitigate against ageism, a pervasive and multifaceted form of discrimination. We describe the rationale for, and context of, the program delivery, before presenting an overview of trainee’s feedback. Thematic analysis of feedback centered around three main themes: knowledge acquisition, change in clinical practice, and enjoyment of the sessions. Trainees (*n* = 20) reported enjoying the sessions, with 100% likely to recommend to peers. The focus on underserved groups, the novel content of the sessions, which were not taught elsewhere, and the non-medical perspective of the speakers were described as particularly valuable. By sharing our approach and reflecting on the early success of the program, we argue for an increased focus on Social Gerontology in Geriatric Medicine education.

## Key summary points

**Aim:**

We describe the design, delivery, and evaluation of a Social Gerontology education programme for Geriatric Medicine trainees in the North-West of England.

**Findings:**

Trainees described enjoying the sessions, gaining novel insights into social problems, and how their practice changed as a result.

**Message:**

At a time of increasing population diversity and inequalities in later life, we argue for an increased focus on Social Gerontology in Geriatric Medicine education.

## Introduction

The practice of Geriatric Medicine involves the comprehensive assessment of older people, including functional, psychological, and social aspects of their lives. However, the focus of Geriatric Medicine education tends toward a biomedical approach. Social gerontology—a field which foregrounds how social, cultural, economic, and environmental factors shape the lives of older adults—has historically been overlooked [1]. This paper describes the delivery of a Social Gerontology education program for Geriatric Medicine trainees in the Northwest of England in 2022–23. We begin with the rationale for, and context of, the program delivery, before presenting an overview of trainee’s feedback. In doing so we argue for a more widespread integration of Social Gerontology in Geriatric Medicine education.

## Why should geriatric medicine trainees receive education in social gerontology?

The aim of delivering a Social Gerontology curriculum to Geriatric Medicine trainees is rooted in the idea that increasing clinicians’ expertise in the social domains of aging will enhance the quality of care provided to older people. Several gerontologists have drawn on evidence to advocate for this integration, often citing the impact Social Gerontology education might have on ageism [1, 2]. Ageism is a pervasive form of discrimination and in contemporary society ageism intersects with other forms of discrimination, such as homophobia and racism, to impact older people differentially [3]. Education that deepens understanding of the events, values, and experiences that shape older patients' lives can improve attitudes toward older people. [4, 5] As population inequalities and diversity increase, Social Gerontology can help clinicians understand the heterogeneity within older populations, how this is influenced by economic and socio-political factors, and how this impacts health [6, 7]. Although programs overhauling undergraduate programs have included a focus on ageism and postgraduate qualifications in Social Gerontology are available in the UK, we were unable to find any examples of Social Gerontology in postgraduate clinical training [8, 9].

## Developing and delivering a social gerontology education program to geriatric medicine trainees

The delivery of teaching for Specialty Trainees is variable across specialties and regions. In the Northwest, all doctors undertaking Specialty Training in Geriatric Medicine in the North-Western Deanery are expected to attend 16 compulsory training days annually. Since 2001, the teaching day program has run in parallel with a Masters in Geriatric Medicine in partnership with the University of Salford. The Masters was developed to provide closer adherence to the training curriculum, an enhanced learning framework, and more robust academic rigor. Some trainees opt to be assessed in specific modules, progressing to a dissertation and a full Masters award, but this is not compulsory. Modules are varied, with a tendency to be taught through a more clinical and biological lens, although content also includes allied domains including medical ethics and law, clinical leadership skills, and medical education.

The authors designed the Social Gerontology program in 2022. LT selected content around the core domains of outlined by Tinker: demography, sociology of aging, psychology of aging, and social policy [1]. However, given evidence around the intersections of disadvantage faced by older people and population inequality and diversity in the Northwest of England, an intersectional approach was used [3, 10]. Intersectionality recognizes how various forms of discrimination and oppression intersect and interact, affecting individuals who hold multiple marginalized identities [11]. The program therefore included sessions on: poverty in later life; aging in place and in cities; social theories of aging; older asylum seekers and refugees; older LGBTQI communities; older people in prison; forensic gerontology and elder abuse. Subject experts from academia, charities, and clinical practice delivered sessions over five in-person training days over the academic year 2022–2023 and were integrated into an existing ‘Gerontology’ MSc module.

Ten trainees opted to be assessed on the module. The assessment was modified from the previous format of written essay on a biomedical aspect of aging, to the production of a research poster on Social Gerontology. The poster was presented to peers and module leaders (authors LT and AMT) on the final in-person training day. Poster subject was chosen by the trainee and included: digital inequalities; resisting ageism; ageism, sexism, and beauty standards; ageism in sexual health; older people in prison; and the transgender community in residential care.

## Trainee’s views of the social gerontology education program

20 of 42 trainees provided responses to an anonymous online feedback survey, distributed by email and WhatsApp, in June 2023. The survey consisted of five Multiple Choice Questions (MCQs) and seven free text responses. Questions focused on the quality and impact of the sessions. Thematic analysis of the qualitative data was performed by LT, generating three core themes: (i) Knowledge gained, (ii) change in practice; and (iii) enjoyment [12]. Results are presented around these themes with illustrative quotes in *‘italics’* and accompanying descriptive statistics.

### I. Knowledge acquisition

80% (*n* = 16) of respondents reported gaining ‘a great deal’ of knowledge from Social Gerontology sessions. 15% (*n* = 3) reported gaining 'a lot', and 5% (*n* = 1) reported gaining some knowledge. Trainees described how speakers from the voluntary sector offered broader perspectives. The content was frequently described as being novel, but useful:

*‘Equips you with the skills that cannot be learned from books alone’*

*‘Forensic gerontology and Elder Abuse—not taught elsewhere, very relevant, the type of problem Elderly Medicine Consultants will be asked to lead on, without having formal training’*

*‘I think these sessions add a massive amount to our education. It’s refreshing to hear non-medical perspectives and crucial to grounding our discussion of older people’s health in Greater Manchester’s economic, population and cultural reality.’*

Trainees described how this allowed them to reframe their approaches to addressing certain issues.

### II. Relevance to work and change in practice

70% (*n* = 14) of respondents reported that the Social Gerontology sessions resulted in influenced their clinical practice. A minority (25%, *n* = 5) expressed uncertainty, while one (5%, *n* = 1) stated that the sessions had not influenced their practice.

Trainees reported adjusting their clinical practice when treating vulnerable patients. Several described changing consultation styles to include more inclusive language and a consideration of wider determinants of health. Specifically, understanding experiences of refugees, older LGBTQ + people, and older people in prisons prompted practice changes:

*‘I’m more likely to push for equity and probe extended reasons for poor health and repeated presentations with the knowledge in mind from these sessions. I am more likely to get involved in community Geriatrics … and more likely to be an activist, which we all should be.’*

*‘I feel like it will have shaped my consultant practice (before it’s even started) as this is where I feel you may have more scope to enact meaningful change/improvement.’*

Some feedback suggested that the Social Gerontology sessions highlighted areas of inequality, and provided insights on how to address this, while one trainee asked for further sessions on* ‘what we can practically do to support patients’.*

### III. Enjoyment

85% (*n* = 17) of respondents described enjoying the Social Gerontology sessions a ‘great deal’. 100% (20) said they would recommend the sessions to other geriatricians. Trainees particularly valued the variety of topics and the focus on underserved populations:

*‘The [LGBTQ*+*] talks were extremely helpful and instructive and it was really heartwarming to be surrounded by speakers (and the other trainees) who were so engaged with these sessions and the needs of older LGBTQ*+ *people’*

Several trainees have contacted LT about undertaking the Masters dissertation in Social Gerontology.

## Critical reflections and next steps

There are limitations to both the program implementation and the feedback presented. The limited time available meant that speakers delivered sessions on often-substantial Social Gerontology subjects in just 25 min. This risked providing a cursory overview of complex issues; indeed, the group discussions which followed often spilled over the allotted time. The feedback survey has a small sample size with vulnerability to selection and social desirability bias. Although the survey was anonymous, trainees knew the feedback would be read by the module leads and trainees who enjoyed the sessions may be more likely to respond. Some trainees wanted a more practical focus for the sessions, feedback which will be incorporated into the program in future.

Despite these limitations, we believe that in a contemporary context of increasing inequalities in wealth and health, and an increasingly diverse older population, there is a renewed case for integrating Social Gerontology into Geriatric Medicine education. This is an area worthy of academic attention. We have identified a pathway to impact, as the trainees’ feedback suggested, Social Gerontology education might change their attitudes and practice. Future research should seek evidence of that impact, and the extent to which Social Gerontology education improves the quality of care for older people. Such research might evaluate trainees’ clinical practice’s pre- and post-educational intervention, and could involve feedback from multidisciplinary colleagues and older people themselves. We believe this program is the first of its kind to be described in the literature. [13] In sharing both how we implemented this initiative, and the feedback received, we hope others might adopt and develop training of a similar scope and focus.
